# Rosiglitazone, a Ligand to PPAR*γ*, Improves Blood Pressure and Vascular Function through Renin-Angiotensin System Regulation

**DOI:** 10.1155/2019/1371758

**Published:** 2019-02-03

**Authors:** María Sánchez-Aguilar, Luz Ibarra-Lara, Leonardo Del Valle-Mondragón, María Esther Rubio-Ruiz, Alicia G. Aguilar-Navarro, Absalom Zamorano-Carrillo, Margarita del Carmen Ramírez-Ortega, Gustavo Pastelín-Hernández, Alicia Sánchez-Mendoza

**Affiliations:** ^1^Department of Pharmacology, Instituto Nacional de Cardiología Ignacio Chávez, Juan Badiano No. 1, Col Sección XVI, Tlalpan, 14080 México, Ciudad de México, Mexico; ^2^Department of Molecular Medicine, Laboratory of Computational Biophysics and Biochemistry, ENMH-IPN, Guillermo Massieu Helguera No. 239, Fracc. La Escalera Ticoman, Gustavo A. Madero, 07320, Ciudad de México, Mexico; ^3^Department of Physiology, Instituto Nacional de Cardiología Ignacio Chávez, Juan Badiano No. 1, Col Sección XVI, Tlalpan, 14080 México, Ciudad de México, Mexico

## Abstract

Rosiglitazone (RGZ), a peroxisome proliferator-activated receptor gamma (PPAR*γ*) ligand, has been reported to act as insulin sensitizer and exert cardiovascular actions. In this work, we hypothesized that RGZ exerts a PPAR*γ*–dependent regulation of blood pressure through modulation of angiotensin-converting enzyme (ACE)-type 2 (ACE2)/angiotensin-(1-7)/angiotensin II type-2 receptor (AT_2_R) axis in an experimental model of high blood pressure. We carried on experiments in normotensive (Sham) and aortic coarctation (AoCo)-induced hypertensive male Wistar rats. Both sham and AoCo rats were treated 7 days with vehicle (V), RGZ (5 mg/kg/day), or RGZ+BADGE (120 mg/kg/day) post-coarctation. We measured blood pressure and vascular reactivity on aortic rings, as well as the expression of renin-angiotensin system (RAS) proteins. We found that RGZ treatment in AoCo group decreases blood pressure values and improves vascular response to acetylcholine, both parameters dependent on PPAR*γ*-stimulation. RGZ lowered serum angiotensin II (AngII) but increased Ang-(1-7) levels. It also decreased 8-hydroxy-2′-deoxyguanosine (8-OH-2dG), malondialdehyde (MDA), and improved the antioxidant capacity. Regarding protein expression of RAS, RGZ decreases ACE and angiotensin II type 1 receptor (AT_1_R) and improved ACE2, AT_2_R, and Mas receptor in AoCo rats. Additionally, an* in silico* analysis revealed that 5′UTR regions of RAS and PPAR*γ* share motifs with a transcriptional regulatory role. We conclude that RGZ lowers blood pressure values by increasing the expression of RAS axis proteins ACE2 and AT_2_R, decreasing the levels of AngII and increasing levels of Ang-(1-7) in a PPAR*γ*-dependent manner. The* in silico* analysis is a valuable tool to predict the interaction between PPAR*γ* and RAS.

## 1. Introduction

Pathological changes occurring in arterial hypertension are common to cardiovascular disease and endothelial dysfunction development. A key player in the pathophysiology of arterial hypertension is angiotensin II (AngII), the main component of the renin-angiotensin system (RAS) [1]. AngII exerts its actions through the interaction with AngII type 1 (AT_1_R) and type 2 (AT_2_R) receptors, producing opposite effects upon stimulation; AT_1_R mediates vasoconstriction, hypertrophy, and rise in reactive oxygen species (ROS) formation, while AT_2_R has been related to vasodilator and antiproliferative actions [2].

The participation of peroxisome proliferator-activated receptors (PPARs) modulating arterial blood pressure has also been documented [3]. PPARs are highly relevant nuclear receptors involved in a variety of physiological processes. Up to date, three isoforms have been described *α*, *β*/*δ*, and *γ*. They present tissue differential distribution and function as transcription factors. PPAR*α* is localized in liver, muscle, kidney, heart, and vessels and participates in fatty acid metabolism [4]. PPAR*γ* can be found in adipose tissue and muscle and it is involved in glucose metabolism and adipocytes differentiation [5, 6]. PPAR*β*/*δ* is ubiquitous and exerts neuroprotective and anti-inflammatory actions [5]. The relationship between PPARs and cardiovascular diseases has been proposed by several research groups. Barroso et al. reported that a single nucleotide mutation (P467L or V290M) in the ligand binding domain of PPAR*γ* is present in subjects with diabetes mellitus and hypertension, suggesting that an alteration in this receptor is important in the control of insulin sensitivity and blood pressure and the presence of this mutation predisposes to disease [7].

It is known that PPAR*γ* activation occurs upon binding to specific ligands. These ligands may be endogenous, like 15-deoxi-Δ^12,14^-prostaglandine J_2_ or exogenous such as thiazolidinediones [TZD, e.g., pioglitazone and rosiglitazone (RGZ)]. Although these drugs are used as hypoglycemic agents in patients with diabetes mellitus (DM), TZD have been reported to exert a blood pressure lowering effect [8, 9]. Takeda et al. stated that PPAR*γ*-activation decreased AT_1_R expression and lowered blood pressure [10]. Although the effect of RZG on other components of RAS has not been addressed, evidences obtained from* in silico* analysis show the presence of a shared motif in UTRs from RAS genes and PPAR*γ* gene, suggesting that the transcriptional machinery could be activated by RZG. Therefore, we hypothesized that RGZ would exert a blood pressure lowering effect through ACE2/Ang-(1-7)/AT_2_R pathway in a PPAR*γ*-dependent manner.

## 2. Methods

### 2.1. Animals

Male Wistar rats (200 to 250 g) bred and raised in our facilities at National Institute of Cardiology Ignacio Chávez were used for all experiments. They were fed with standard chow diet (Lab Diet 5001, PMI Nutrition International, Brentwood, MO, USA) and the experiments were completed in accordance with the Guide for the Care and Use of Laboratory Animals (SAGARPA, NOM-062-ZOO-1999, Mexico) and by Institutional Ethics Committee.

### 2.2. Experimental Design

Rats were randomly distributed into five groups: (1) Sham-ligated vehicle-administrated (Sham-V), (2) Sham-ligated rosiglitazone treated (Sham-RGZ) [5 mg/kg, intraperitoneally (I.P.), (Cayman Chemical, Ann Arbor, MI, USA)], (3) aortic coarctation (AoCo)-vehicle (AoCo-V), (4) AoCo-rosiglitazone (AoCo-RGZ), and (5) AoCo-RGZ plus bisphenol A diglycidyl ether [(BADGE), (AoCo-RGZ+BADGE) (120 mg/kg, I.P.; Sigma Aldrich, St. Louis, MO, USA)]. Treatment was applied immediately after surgery and every 24 h for 7 days. At the end of the treatment, rats were anaesthetized [50mg/Kg sodium pentobarbital, I.P., (Pfizer, S.A. de C.V., México)] and the blood pressure was measured. Later, we collected and centrifuged blood samples at room temperature (RT, at 3000 x* g*) to obtain serum and it was stored at -70°C to be used for the measurement of total antioxidant capacity (TAC), malondialdehyde (MDA), 8-hydroxy-2′-deoxyguanosine (8-OH-2dG), AngII, and Ang-(1-7). At the same time, we obtained tissue samples to evaluate protein expressions.

#### 2.2.1. Aortic Coarctation Surgery

Experimental animals underwent sham or AoCo surgery. AoCo-induced hypertension was performed under anesthesia with isofluorane (Laboratorios PiSA, Guadalajara, Jalisco, México). We carried out abdominal laparatomy to expose the peritoneum, localize the abdominal aorta, and either make a false aortic ligation (Sham) or partially ligate the artery with silk (3-0) at inter-renal level (AoCo). With this maneuver, we reduced approximately 75% of the blood flow to the distal part. Rats were sutured by layers and allowed to recover [11]. Treatment was applied immediately after and every 24 h for 7 days. Neither the surgery nor pharmacological treatment modified body weight gain or food intake. At the end of the treatment, the blood pressure was measured.

#### 2.2.2. Blood Pressure Measurement

Arterial blood pressure was recorded intracarotid, with a blood pressure transducer coupled to a blood pressure monitor (BP-1, World Precision Instruments, Sarasota, FL, USA), seven days after surgery. Rats were anesthetized with sodium pentobarbital (50 mg/kg, I.P.) and an incision was performed in the neck. Carotid artery dissection was carried out to introduce a polyethylene cannula (50 *μ*m, PE-50) containing a heparinized solution (10%), connected to a BP-1 monitor and to an acquisition system (Duo 18, World Precision Instruments, Sarasota, FL, USA). The values were consistently acquired between 9:00 and 10:00 AM. Systolic blood pressure was recorded for ten minutes and reported as the mean ± standard error of the mean (S.E.M.) of 6 different experiments [12].

#### 2.2.3. Determination of Vascular Reactivity

Thoracic aortas were obtained immediately after sacrifice and maintained in Tyrode solution (140 mM NaCl, 5 mM KCl, 1 mM CaCl_2_, 1 mM MgCl_2_, 5 mM HEPES, and 5.5 mM glucose, all reagents were purchased from Sigma Aldrich, St. Louis MO, USA) pH 7.4, 37°C and oxygenated (95% O_2_ – 5% CO_2_). Fat and connective tissue were removed under stereoscopic microscope. Concentration-response curves to acetylcholine (ACh, Sigma Aldrich, St. Louis MO, USA) 1x10^−10^ to 1x10^−5^ M on aortic rings were obtained from every experimental group under basal conditions and in presence of 300 *μ*M of N^G-^ nitro L-arginine methyl ester (L-NAME, Sigma Aldrich, St. Louis MO, USA) to study nitric oxide-dependent vasorelaxant response [13].

#### 2.2.4. Protein Expression by Western Blot

Total protein content in aortas was quantified using bicinchoninic acid assay (Thermo Scientific, Rockford IL, USA). Protein extracts (100 *μ*g) were resolved on 10-12% SDS-PAGE, blotted onto polyvinylidene fluoride (PVDF) membranes (0.45 *μ*m, Millipore, Billerica MA, USA), and then blocked with 5% non-fat milk (Bio-Rad, Hercules CA, USA) in PBS-0.1% tween as reported elsewhere [14]. The membranes were incubated overnight at 4°C with primary antibodies against *β*-actin (1:5000), AT_1_- and AT_2_-receptors (1:1000), ACE and ACE2 (1:2500), and Mas receptor (1:100) (Santa Cruz Biotechnology, Santa Cruz, CA, USA) diluted in 1% non-fat milk. Later, membranes were washed and incubated with secondary antibodies (dilution 1:5000, Jackson ImmunoResearch, West Grove, PA, USA) and proteins were developed with chemiluminescent HRP substrate (Immobilon Western, Millipore, Billerica MA, USA) [14].

#### 2.2.5. Quantification of AngII and Ang-(1-7) in Biological Samples

On the day of analysis, samples were gradually thawed at 1-hour intervals, first at -50°C, -20°C, -10°C, and finally at a temperature below 20°C. Each thawed sample (200 *μ*L) was deproteinized with 1 mL of cold methanol, 20% trichloroacetic acid (10:1) and centrifuged at 16000 x* g*, 15 min at 4°C. The supernatants were filtered through nitrocellulose membranes (0.22 *μ*m), diluted (1:10) with 0.1 N NaOH, and passed through Sep-Pak classic C-18 cartridge (Waters Corporation, Milford, MA, USA). Finally, angiotensins were analyzed by capillary electrophoresis (P/ACE MDQ Capillary Electrophoresis System, Beckman Coulter, CA, USA). The preconditioning of the capillary was done with a solution of 1 N NaOH, deionized water, and finally running buffer (100 mM boric acid and 3 mM tartaric acid, pH 10) for 30 minutes. The sample was injected under hydrodynamic pressure of 0.5 psi/10 s. The separation was done at 30 kV for 10 minutes at 200 nm and 10°C. The concentrations were obtained interpolating values with standard curves of AngII and Ang-(1-7) (both from Sigma Aldrich, St. Louis, MO, USA), obtained under the same conditions as the samples. Data is expressed as pmol/mL [15, 16].

#### 2.2.6. Quantification of MDA in Serological Samples

Serum samples (50 *μ*L) were deproteinized with cold methanol (1:1 v/v) and centrifuged at 16000 x* g* for 15 min at 10°C. The supernatants (80 *μ*L) were filtered with a nitrocellulose membrane (0.22 *μ*m, Millipore, Billerica MA, USA) and diluted (10:1) with 0.1 N NaOH. Finally, samples were analyzed by capillary electrophoresis (P/ACE MDQ Capillary Electrophoresis System, Beckman Coulter, CA, USA) following a previously reported protocol. The concentrations were obtained interpolating from standard curves of MDA (Sigma Aldrich, St. Louis, MO, USA) which were treated in the same conditions as the samples [17].

#### 2.2.7. Quantification of 8-OH-2-dG in Biological Samples

The marker of DNA damage, 8-OH-2-dG, was quantified in aortic and serum samples. The samples (50 *μ*L) were deproteinized with cold methanol (1:5 v/v) and centrifuged at 16000 x* g *for 15 min at 10°C. The supernatants were filtered through a Sep-Pak classic C-18 cartridge (Waters Corporation, Milford, MA, USA) and mixed with 10 *μ*L of perchloric acid 5% (1:5). The samples were filtered through a nitrocellulose membrane (0.22 *μ*m), diluted with 50 mM sodium phosphate monobasic at pH 7.4 (1:10), and analyzed by capillary electrophoresis (P/ACE MDQ Capillary Electrophoresis System, Beckman Coulter, CA, USA). The conditions of injection were taken from Kvasnicová reference [18].

#### 2.2.8. Antioxidant Capacity Assay

Total antioxidant capacity was determined on serum samples. We placed 35 *μ*L of serum in a 96-well plate. Later, we added 145 *μ*L of 0.1 M phosphate buffer at pH 7.5 and shook this to 500 rpm for 200 sec. We transferred 100 *μ*L of diluted serum to the adjacent well and added 50 *μ*L of 0.01 M CuCl_2_ and shook at 500 rpm for 200 sec. Then we added 50 *μ*L of 0.01 M batocuproin and vortexed again at 500 rpm for 200 sec. Samples obtained were quantified by spectrometry to 490 nm (DW2000, SLM-Amico, Urbana, IL, USA). Total antioxidant capacity is expressed as *μ*M of Cu^2+^ reduced to Cu^+^ [19].

#### 2.2.9. PPAR*γ* Transcription Factor Activity Assay

The assay measures PPAR*γ* binding activity in nuclear extract. It was measured in aortic tissue. Aortic nuclear fraction was obtained using NE-PER extraction reagents kit (Thermo Scientific, Rockford, IL, USA). Later, nuclear protein obtained was incubated in ELISA plate, containing PPAR response element (PPRE)-DNA sequence, following the instructions from manufacturer (PPAR*γ* transcription factor assay kit, Cayman Chemicals, Ann Arbor, MI, USA). The colored product was spectrophotometrically measured at 450 nm. The PPAR*γ* activity is expressed as absorbance/mg of protein quantified.

#### 2.2.10. *In Silico *Methodology

We obtained 5′UTR nucleotide sequences belonging to RAS pathway (*ACE, ACE2, renin, angiotensinogen, AT*_*1*_*R, AT*_*2*_*R*, and* Mas receptor*) and* PPARγ* genes of the rat genome from Ensemble database (NCBI) (https://www.ncbi.nlm.nih.gov/gene/) and built a FASTA file. The file was analyzed using the Multiple Em for Motif Elicitation program (MEME) (version. 2013) (meme-suite.org) to identify patterns of sequence called motifs. This program was used with website predetermined conditions. Later, these motifs were screened in the Transcription Element Search System (TESS) database (version. 2013) (www.cbil.upenn.edu/cgi-bin/tess/tess) to be associated with transcriptional factors previously reported. Quantification of nucleotide percentage in the motifs was also performed [20].

#### 2.2.11. Statistical Analysis

Statistical analyses were carried out using GraphPad Prism 5 software. The results are expressed as the mean ± standard error of the mean. For multiple comparisons, we used one-way analysis of variance (ANOVA) followed by* post hoc* (Tukey). Statistical differences were considered when the* P *value was <0.05.

## 3. Results

### 3.1. Rosiglitazone Lowers AoCo-Induced Hypertension

Seven days after AoCo surgery, we measured systolic blood pressure in carotid artery. Rats from AoCo-V group showed a higher systolic blood pressure compared to Sham-V (154.2±3.5 vs. 112.3±3.3 mmHg, respectively,* P* < 0.05). RGZ prevented the increase in arterial blood pressure elicited by AoCo (136.3±2.1 mmHg,* P* < 0.05). The RGZ-induced antihypertensive effect was blunted by BADGE (160.3±3.7 mmHg,* P* < 0.05), a PPAR*γ* antagonist, suggesting the participation of the receptor regulating blood pressure.

### 3.2. Rosiglitazone Improves Vascular Responses

**Figure 1:**
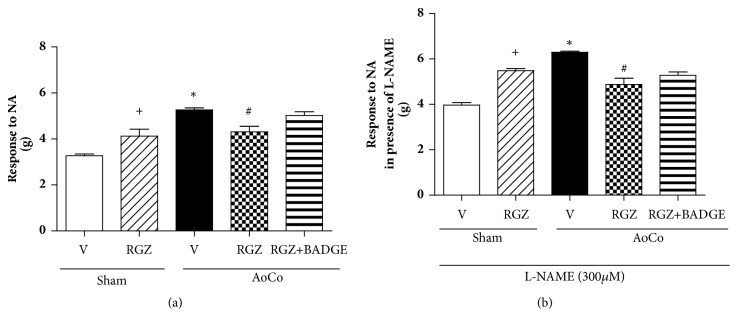
Effect of rosiglitazone (RGZ) on noradrenaline (NA)-induced aortic reactivity in absence (a) or presence (b) of N(*ω*)-nitro-L-arginine-methyl-ester (L-NAME, 300*μ*M). The values represent the mean ± SEM (n=6 animals per group). One-way analysis of variance (ANOVA) post Tukey. ^+^*P *< 0.05 sham-V vs. sham-RGZ, ^∗^*P* < 0.05 sham-V vs. AoCo-V, ^#^*P* < 0.05 AoCo-V vs. AoCo-RGZ.

Regarding contractile response to NA in basal conditions, aortic rings from AoCo-vehicle rats showed a higher NA-induced vasoconstriction compared to sham-vehicle (*P* < 0.05). Rosiglitazone treatment decreased NA-induced vascular response in AoCo's aortic rings compared to those from AoCo-V (*P* < 0.05). AoCo-RGZ+BADGE did not modify the NA-induced vasoconstrictor effect (Figure 1(a)). This response was evaluated also in presence of L-NAME, an inhibitor of NO production. Our results show a higher vasoconstriction on AoCo-vehicle group compared with sham-vehicle group (*P* < 0.05) and RGZ treatment was capable of diminishing NA-induced vasocontractile response (*P* < 0.05). Once again vascular responses to NA on aortic rings from AoCo-RGZ+BADGE were not different from AoCo-RGZ treated rats (Figure 1(b)).

**Figure 2:**
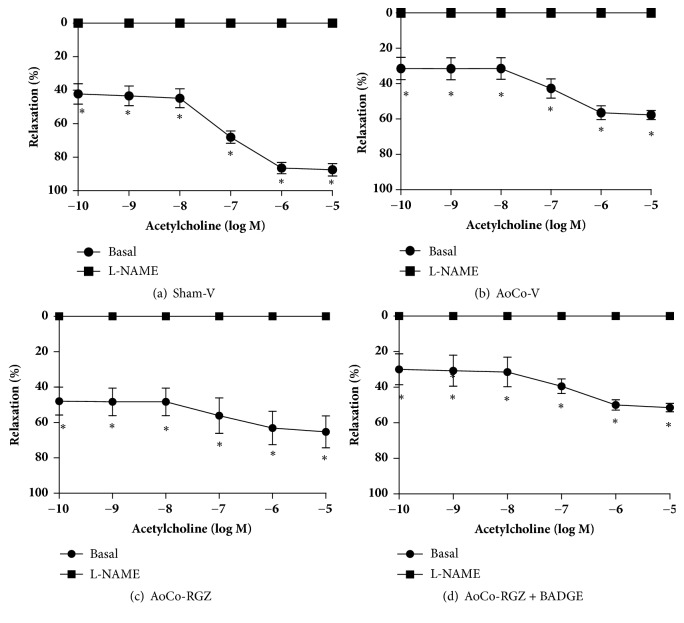
Effect of rosiglitazone (RGZ) on vascular reactivity exerted by acethylcholine. The aortic reactivity was evaluated in sham-vehicle treated (a), aortic coarctated (AoCo)-vehicle treated (b), AoCo-RGZ treated (c), and AoCo-RGZ + bisphenol A diglycidyl ether (BADGE) treated (d) rats under basal conditions (●) or in presence of N(*ω*)-nitro-L-arginine-methyl-ester (L-NAME, 300*μ*M) (■). The values represent the mean ± SEM (n=6 animals per group). One-way analysis of variance (ANOVA) post Tukey. ^∗^*P* < 0.05 basal vs. L-NAME conditions.

Concentration-response curves to ACh (1x10^−10^ to 1x10^−5^ M) were performed in precontracted aortic rings [NA (1x10^−6^ M)]. Sham-V rats showed a concentration-dependent response to ACh. Aortic rings from AoCo-V group exhibited a lower ACh-induced vasorelaxant response compared with Sham-V group (*P* < 0.05). Aortic rings from AoCo rats treated with RGZ displayed an improvement in ACh-induced relaxation compared to vehicle-treated hypertensive rats. The use of BADGE tended to prevent the RGZ effect. Vasorelaxant response to ACh was abolished in presence of L-NAME (*P* < 0.05) suggesting that RGZ-induced vasorelaxation was, in every case, dependent on NO (Figures 2(a), 2(b), 2(c), and 2(d)).

### 3.3. Effect of Rosiglitazone on RAS Axis in Hypertensive Rats

**Figure 3:**
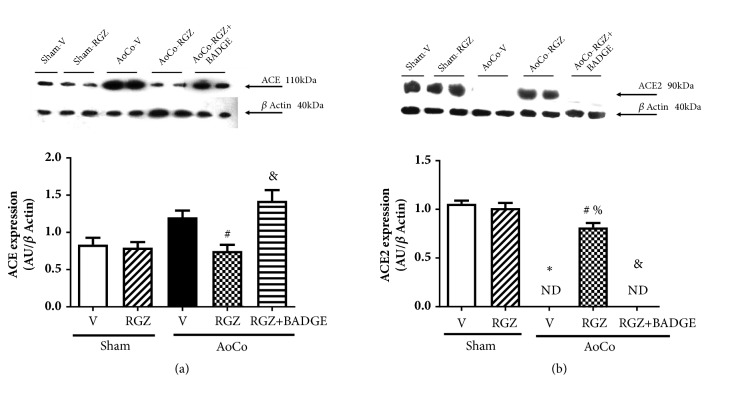
Effect of rosiglitazone (RGZ) on aortic angiotensin converting enzyme (ACE) (a) and ACE2 (b) expression. The images show representative western blots. The protein expression was evaluated in homogenate of aorta from sham or aortic coarctated (AoCo) rats treated with vehicle (V), rosiglitazone (5mg/kg, RGZ), or RGZ + bisphenol A diglycidyl ether (120 mg/kg, RGZ+BADGE). The values represent the mean ± SEM (n=6 animals per group). One-way analysis of variance (ANOVA) post Tukey. ^∗^*P* < 0.05 Sham-V vs. AoCo-V, ^#^*P* < 0.05 AoCo-V vs. AoCo-RGZ, ^%^*P* < 0.05 Sham-RGZ vs. AoCo-RGZ, and ^&^*P* < 0.05 AoCo-RGZ vs. AoCo-RGZ+BADGE. ND: not detected.

To determine if RGZ exerts a regulatory effect on RAS components, we evaluated the expression of proteins conforming RAS pathway. Tisular and serum levels of AngII and Ang-(1-7) were also quantified. We found that ACE, in aortas from AoCo-RGZ rats, exhibits a lower expression compared to AoCo-V (*P* < 0.05), an effect that was absent in the arteries from AoCo-RGZ+BADGE-treated subjects (*P* < 0.05) (Figure 3(a)). Regarding ACE2, for AoCo-V group the expression of this enzyme was undetectable (*P* < 0.05), while RGZ augmented significantly its expression (*P* < 0.05) and BADGE reverted the RGZ-induced effect (*P* < 0.05) (Figure 3(b)).

**Figure 4:**
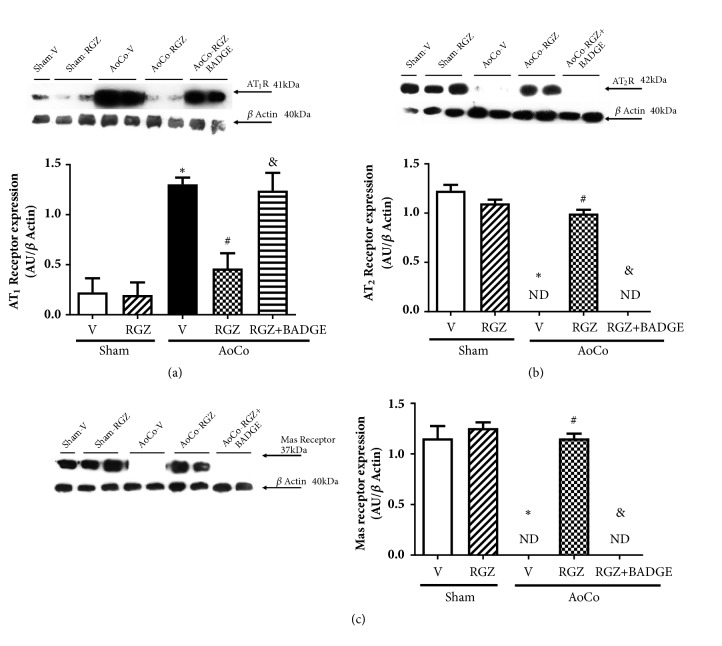
Effect of rosiglitazone (RGZ) on aortic angiotensin II type 1 (AT_1_R) (a), angiotensin II type 2 (AT_2_R) (b), and Mas (c) receptor expression. The images show representative western blots. The protein expression was evaluated in homogenate of aorta from sham or aortic coarctated (AoCo) rats treated with vehicle (V), rosiglitazone (5mg/kg, RGZ), or RGZ + bisphenol A diglycidyl ether (120 mg/kg, RGZ+BADGE). The values represent the mean ± SEM (n=6 animals per group). One-way analysis of variance (ANOVA) post Tukey. ^∗^*P* < 0.05 Sham-V vs. AoCo-V, ^#^*P* < 0.05 AoCo-V vs. AoCo-RGZ, and ^&^P < 0.05 AoCo-RGZ vs. AoCo-RGZ+BADGE. ND: not detected.

On the other hand, we evaluated the AngII receptors: AT_1_ and AT_2_. In the case of AT_1_, we found that the expression of this receptor in hypertensive rats receiving vehicle was augmented (*P* < 0.05) and RGZ decreased the AoCo-induced expression compared with vehicle (*P* < 0.05). The AoCo-RGZ+BADGE reverted this effect (*P* < 0.05) (Figure 4(a)). With respect to AT_2_, in hypertensive group, the expression of this protein was not detected (*P* < 0.05) but RGZ treatment promoted an increment compared to AoCo-V (*P* < 0.05). The administration of BADGE prevented the beneficial effect obtained in AoCo-RGZ (Figure 4(b)). In order to explore the participation of Mas receptor, we evaluated its expression, AoCo promoted a fall in Mas receptor tissue content, and as expected, RGZ promoted recovery of its expression, an effect prevented by BADGE in AoCo-RZG treated rats (*P* < 0.05) (Figure 4(c)).

**Figure 5:**
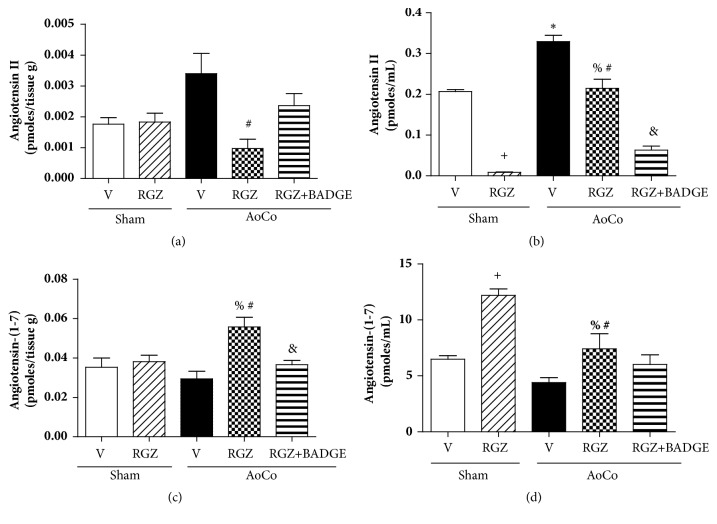
Effect of rosiglitazone (RGZ) on angiotensin II and angiotensin-(1-7) production. Values obtained from aorta's homogenate (a, c) and serum (b, d) from sham or aortic coarctated (AoCo) rats treated with vehicle (V), rosiglitazone (5mg/kg, RGZ), or RGZ + bisphenol A diglycidyl ether (120 mg/kg, RGZ+BADGE). The values represent the mean ± SEM (n=6 animals per group). One-way analysis of variance (ANOVA) post Tukey. ^∗^*P* < 0.05 Sham-V vs. AoCo-V, ^+^*P* < 0.05 Sham-V vs. Sham-RGZ, ^%^*P* < 0.05 Sham-RGZ vs. AoCo-RGZ, ^#^P < 0.05 AoCo-V vs. AoCo-RGZ, and ^&^*P* < 0.05 AoCo-RGZ vs. AoCo-RGZ+BADGE.

With respect to AngII concentration, we observed that AoCo shows a tendency to increase this tissue peptide level and RGZ treatment decreases this level significantly (*P* < 0.05). On the other hand, AoCo promoted an increase in serum (*P* < 0.05), RGZ again decreased the level of this peptide (*P* < 0.05), and RGZ+BADGE further decreased AngII concentration (*P* < 0.05) (Figures 5(a) and 5(b)).

We also measured aortic and serum concentration of Ang-(1-7). In both samples, RGZ increased significantly Ang-(1-7) compared with vehicle (*P* < 0.05), and AoCo induced a fall in Ang-(1-7) serum concentration, an effect that was prevented by RGZ treatment (*P* < 0.05). In tissue, BADGE prevented RGZ-induced effect, while in serum it exerted no further effect (Figures 5(c) and 5(d)).

### 3.4. Rosiglitazone Improves Redox Balance in Hypertensive Rats

**Figure 6:**
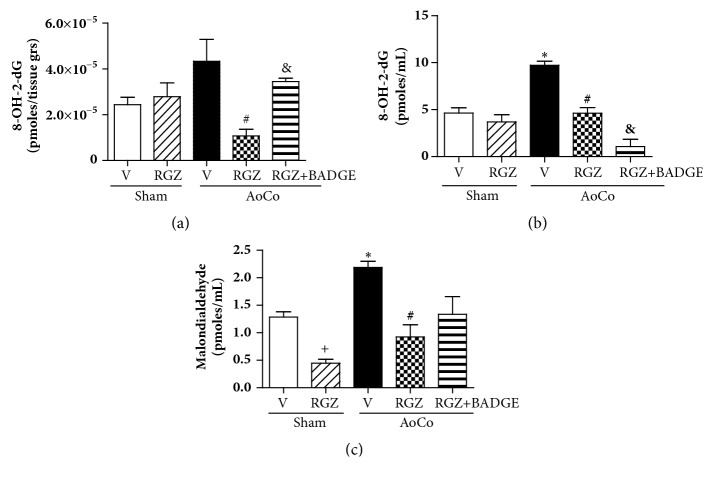
Effect of rosiglitazone (RGZ) on 8-hydroxy-2′-deoxyguanosine (8-OH-2-dG), a marker of oxidative stress-induced DNA damage in homogenate from aorta (a) and serum (b), and serum malondialdehyde (c) from sham or aortic coarctated (AoCo) rats treated with vehicle (V), rosiglitazone (5mg/kg, RGZ), or RGZ + bisphenol A diglycidyl ether (120 mg/kg, RGZ+BADGE). The values represent the mean ± SEM (n=6 animals of group). One-way analysis of variance (ANOVA) post Tukey. ^∗^*P* < 0.05 Sham-V vs. AoCo-V, ^#^*P* < 0.05 AoCo-V vs. AoCo-RGZ, and ^&^*P* < 0.05 AoCo-RGZ vs. AoCo-RGZ+BADGE.

In order to explore the role of RGZ on oxidant-antioxidant system, we quantified 8-OH-2dG, a marker of ROS-induced DNA damage in both tissue and serum samples. We found that tissue from AoCo rats shows a slightly higher content of 8-OH-2dG compared to Sham-V and RGZ was capable of decreasing it (*P* < 0.05). The coadministration of BADGE to AoCo-RZG rats reverted the antioxidant effect (*P* < 0.05) (Figure 6(a)). Data obtained from serum analysis shows that AoCo raised 8-OH-2dG (*P* < 0.05). On the other hand, RGZ was able to reduce the values with respect to AoCo-V (*P* < 0.05). Surprisingly in serum, AoCo-RGZ+BADGE behaved in a RGZ-like manner preventing oxidative stress (*P *< 0.05) (Figure 6(b)).

Additionally, we measured MDA, a marker of lipid oxidation. We show that AoCo induced an increased level of this marker (*P* < 0.05). RGZ treatment in AoCo rats was capable of decreasing this marker. On the other hand, BADGE did not modify RGZ-induced effect (Figure 6(c)).

**Figure 7:**
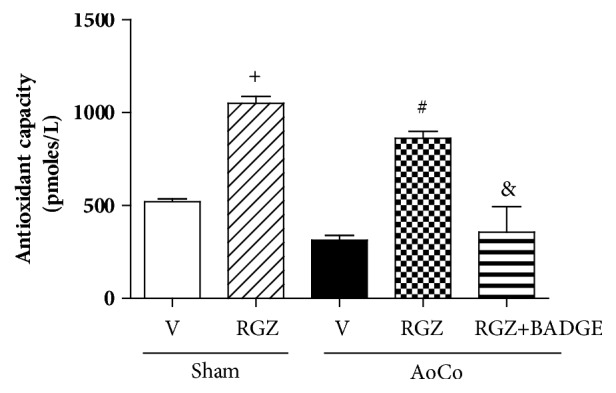
Effect of rosiglitazone (RGZ) on total antioxidant capacity of sham or aortic coarctated (AoCo) rats treated with vehicle (V), rosiglitazone (5mg/kg, RGZ), or RGZ + bisphenol A diglycidyl ether (120 mg/kg, RGZ+BADGE). The parameter was evaluated in serum. The values represent the mean ± SEM (n=6 animals of group). One-way analysis of variance (ANOVA) post Tukey. ^+^*P* < 0.05 Sham-V vs. Sham-RGZ, ^∗^*P* < 0.05 Sham-V vs. AoCo-V, ^#^P < 0.05 AoCo-V vs. AoCo-RGZ, and ^&^*P* < 0.05 AoCo-RGZ vs. AoCo-RGZ+BADGE.

To explore if RGZ modifies serum total antioxidant capacity of the organisms, we evaluated this parameter and our data show that RGZ increases the antioxidant capacity with respect to vehicle treated animals (Sham and AoCo) (*P* < 0.05) and BADGE reverted this effect (*P* < 0.05) (Figure 7).

### 3.5. Rosiglitazone Increases PPAR*γ* Activity

**Figure 8:**
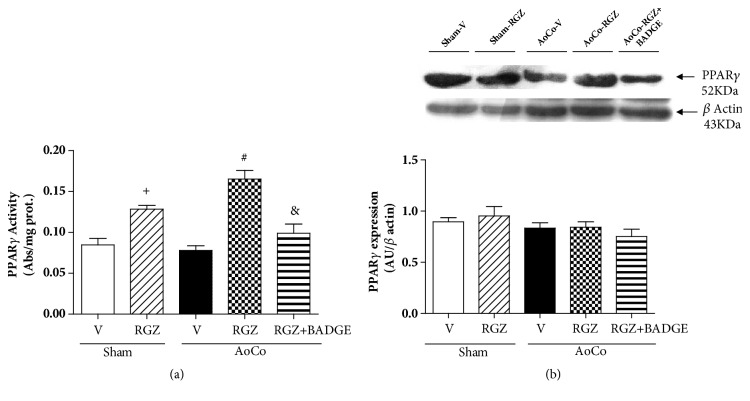
Effect of rosiglitazone (RGZ) on PPAR*γ* activity (a) and PPAR*γ* expression of sham or aortic coarctated (AoCo) rats treated with vehicle (V), rosiglitazone (5mg/kg, RGZ), or RGZ + bisphenol A diglycidyl ether (120 mg/kg, RGZ+BADGE). PPAR*γ* activity was evaluated using a transcription factor assay kit (Cayman Chemicals, Ann Arbor, MI, USA). Western blot image is representative of 6 different experiments. Bars represent the mean ± SEM (n=6 animals of group). One-way analysis of variance (ANOVA) post Tukey. ^+^*P* < 0.05 Sham-V vs. Sham-RGZ, ^#^*P* < 0.05 AoCo-V vs. AoCo-RGZ, and ^&^*P* < 0.05 AoCo-RGZ vs. AoCo-RGZ+BADGE.

In order to explore if the results obtained are dependent on RGZ-induced PPAR*γ*-activation, we evaluated PPAR*γ* transcriptional activity and protein expression. We found that RGZ increased PPAR*γ* activity in Sham rats (*P* < 0.05) and in AoCo group (*P* < 0.05). BADGE prevented RGZ-induced effect on PPAR*γ* transcriptional activity (*P* < 0.05) (Figure 8(a)). Later, we studied if RGZ stimulation modified PPAR*γ* aortic protein expression. As can be observed, there were no changes promoted by RGZ on this parameter (Figure 8(b)).

### 3.6. RAS and PPAR*γ* Genes Share a 5′ UTR Sequence

**Figure 9:**
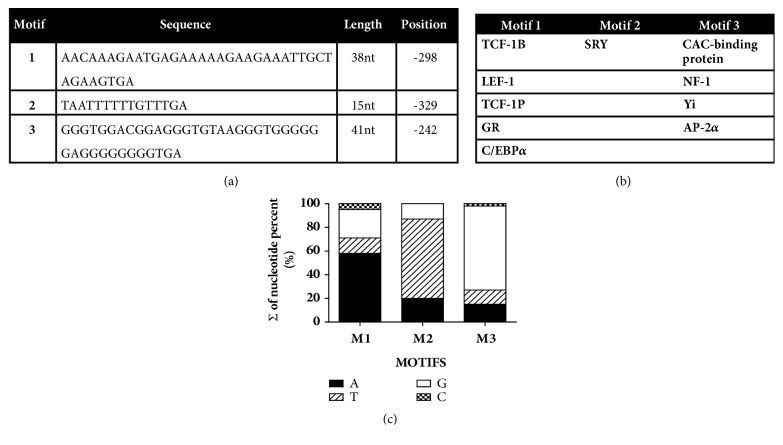
*In silico* analysis obtained by bioinformatic on line tools. The motifs (M) sequences in 5′ untranslated regions obtained by MEME program indicate the length and position regarding the transcription start signal (a). Transcription factors found through TESS database in motifs (b). Percentage of nucleotide presence on these motifs (c).

To get insight into a possible similarity between response elements of RAS and* PPARγ* genes, a bioinformatic analysis was performed using the MEME program. Patterns of sequences were screened in the 5′UTR regions of the following genes: RAS (*ace, ace2, renin, angiotensinogen, at*_1_r*, at*_2_*r,* and* mas receptor*) and* pparγ*. Three shared consensus sequences of nucleotides were detected (Figure 9(a)); one of them is present in every gen studied (GGGTGGACGGAGGGTGTAAGGGTGGGGGGAGGGGGGGGTGA); it is labeled as Motif #3. The lengths of the motifs found were from 15 to 41 nucleotides and they were searched in the TESS database. Motif #1 was localized in the genes of T-cell factor 1B (TCF-1B), lymphoid enhancer factor 1 (LEF-1), T-cell factor 1 [TCF-1(P)], glucocorticoid receptor (GR), and CCAAT/enhancer binding protein alpha (C/EBP*α*), Motif #2 can be localized in testicular factor (SRY), and Motif #3 can be localized in CAC-binding protein, TGGCA union protein (NF-1), Yi factor, and activating enhancer binding protein 2 alpha (AP-2*α*) (Figure 9(b)). Regarding nucleotide frequency per motif, we found that each nucleotide,* id est*, adenine, guanine, and thymine were present in average 30% of motif sequence, while cytosine shows a lower frequency (2.3%) (Figure 9(c)).

## 4. Discussion

In the present study, we have demonstrated that RGZ-induced PPAR*γ*-stimulation exerts a blood pressure lowering effect through activation of RAS vasorelaxant axis, increasing Ang-(1-7) and the expression of AT_2_R and Mas receptor. Additionally, RGZ promotes an antioxidant effect in hypertensive subjects.

Blood pressure lowering effect exerted by RZG, a PPAR*γ* ligand, in an experimental model of high blood pressure secondary to AoCo, could be through RAS vasorelaxant axis. From the begging of RAS discovery (1957) the vasocontractile effect of RAS components has been clearly established. However, later findings demonstrate that RAS includes vasorelaxant components such as Ang-(1-7), AT_2_R, and Mas receptor [21] conforming the recently named: vasorelaxant axis [22].

In our study, the components of this axis [ACE2, AT_2_R, Mas receptor, and Ang-(1-7)] increased in response to RGZ. Moreover, current data also shows that AT_1_R expression decreases, further supporting the hypothesis that PPAR*γ* stimulation lowers blood pressure through modulation of RAS. Previously, Diep QN et al. and Ryan MJ et al. had reported an attenuation of AngII-induced hypertensive effect in response to RGZ or pioglitazone and in a transgenic model of hypertension with RGZ treatment, respectively [8, 23].

Impaired vascular response to several agonists is a commonly found feature in arteries from high blood pressure subjects. For instance, vascular response to endothelin-1 was higher in SHR compared to Wistar-Kyoto control rats [24], young SHRs (6 weeks old) also display an elevated response to AngII, and it is accompanied with structural changes in established phase (21 weeks old) [25]. Angiotensin II enhanced the vasopressor response to alpha adrenergic stimulation; this evidenced in isolated rabbit femoral artery rings [26]. In our experimental model, we studied the vascular response to NA. As expected, NA induced a higher vasoconstriction in arteries from AoCo rats, an effect that was attenuated by RGZ.

We also found that RGZ administration for 7 days to AoCo rats improves the ability of the vessel to dilate in response to ACh, in a NO-dependent manner. These data also imply that PPAR*γ* exerts an antihypertensive effect via NO production, resembling the effect produced by clofibrate (100 mg/kg), a PPAR*α* ligand, reducing blood pressure, and improving vascular reactivity in a NO-dependent manner [27].

The interaction between RAS and PPAR*γ* influences also the production of metabolites. Efrati S et al. reported that the stimulation of PPAR*γ* with RGZ inhibited AngII synthesis in mesangial cells derived from SHR rats [28]. Our results support and extend those of Efrati et al. demonstrating that RGZ decreased the production of AngII in the whole animal, in both aortic and serum samples. We also show that Ang-(1-7), a vasorelaxing component of RAS system, raises in response to RGZ in both sham and AoCo subjects. Our study also points to the relevance of PPAR*γ* modulating AT_1_R, ACE expression, and AngII level, actions that bring relevant consequences for vascular tone and blood pressure. Our group previously reported that PPAR*α* stimulation raises plasma and tissue levels of Ang-(1-7) in an experimental model of AoCo-induced hypertension [15]. In the current study, we hypothesized that Ang-(1-7) increased production is due to RGZ-induced expression of ACE2 in AoCo, an event that could explain also AngII decreased level in AoCo-RGZ rats and vascular response observed.

Although BADGE is considered as a PPAR*γ* antagonist, there are reports affirming that it behaves as an agonist [29, 30]. Our data suggest that BADGE may behave as both. Regarding AngII serum concentration and 8-OH-2dG, BADGE acted as PPAR*γ* agonist. However, it antagonized RGZ-induced effects on blood pressure and expression of tissue proteins (ACE, ACE2, AT_1_R, AT_2_R, Mas receptor). Bishop-Bailey D et al. studying ECV304 cells (bladder carcinoma cells) showed that BADGE induced a raise in PPAR*γ* activity, evaluated by a luciferase gene marker [29]. The authors proposed a tissue specific regulation of PPAR. Another explanation could be that BADGE acts as “biased ligand”, meaning that it produces both agonist and antagonist effects in different tissues or cellular types in the same animal. The biased activity has been reported for *β*2 adrenergic receptor when, stimulated by ligands, it recruits *β*-arrestins [31, 32]. In order to identify the role played by BADGE in our study, we explored the activity of PPAR*γ* as transcription factor in the presence of RGZ and BADGE. The results show that, in our experimental model, BADGE acts as antagonist.

The relationship between PPAR*γ* and RAS components is at genetic level. By means of a bioinformatics analysis, 5′UTR regions present in RAS genes were identified as possible transcriptional regulators. Spontaneo L et al. have described that a sequence motif is a pattern of nucleotides functioning as checkpoints for biological function or regulation [33]; they can be as long as 20 bp, providing a start point to identify transcription factors or nucleotide islands [34]. We found three motifs in 5′UTR sequences; Motif #3 was found on the eight RAS components genes sequences 5′UTR studied. The lengths of the motifs (38, 15, and 41 nucleotides) and their location, at the positions -298, -329, and -242 from the start site of transcription, suggest that they might be part of the recognition and anchoring site of the transcriptional machinery, thereby regulating the expression of target genes.

Another peculiar feature of the motifs was the repeated nucleotide profile. This characteristic has been related to the degree of transcriptional activity. Patel et al. related the presence of a sequence of four guanines in 5′UTR regions of oncogenes with the regulation of telomerase activity of c-Myc oncogene [35]. Also, Iwashina et al. reported that an AU rich sequence in 3′UTR of iNOS mediates cytokine-induced mRNA stabilization [36]. Probably the best example is mRNA polyadenylation, which consists in the addition of adenines to the 3′ extreme stabilizing the mRNA and protecting from RNAses, allowing a greater translation of the target protein [37]. Therefore, it suggests that adenine and guanine sequences in these motifs found in our study could participate as regulators of RAS genes expression.

Regarding transcription factors found in the TESS database, our analysis shows that Motif #1 is contained in the regulatory regions of TCF-1B, TCF-1, and LEF-1(P), which are associated with the regulation and function of the lymphoid lineage. TCF-1B has been reported to suppress IL-17 gene expression, a cytokine associated with the activation and recruitment of monocytes at the inflammation site [38] and being part of the GR, a member of the nuclear receptor superfamily. As is widely reported, GR stimulation exerts an anti-inflammatory effect through NF-kB inhibition [39]; it also participates in the regulation of blood pressure as shown by Goodwin JE who reported that its activation with dexamethasone induced an elevation of blood pressure in mice [40]. Motif #1 could also be part of the mechanism regulating PPAR*γ*. The sequence was found to be part of C/EBP*α*, a factor involved in energy homeostasis, growth, and differentiation of cells and a known regulator of PPAR*γ* [41]. Motif #2 is present in SRY factor, a gender-determining factor. It regulates the activity of tyrosine hydroxylase, an enzyme required for NE synthesis, during vascular responses [42]. The sequence of the Motif #3 was found to be part of* NF-1* gen, codifying for NF-1 proteins (NFI-A, NFI-B, NFI-C and NFI-X) which are expressed in kidney and blood cells, where they participate in the regulation of* α-globin* gene and erythroid cells [43]. Motif #3 is also part of* AP2α* gene; although there is not sufficient information about its function on vascular responses, the presence of AP2*α* recognition sites on heme oxigenase-1 (HO-1) promoter has been reported [44]. Regarding the Yi factor and its role in vascular responses, no information is available. Thus, these transcription factors might be part of a set of modulators participating in signaling pathways shared by RAS- and* PPARγ*-genes.

## 5. Conclusion

Rosiglitazone decreases AoCo-induced rise in systolic blood pressure in a PPAR*γ*-dependent manner. The effect could be due to decreased levels of AngII produced by ACE, an increase in Ang-(1-7) produced by ACE2 as well as augmented expression of AT_2_R and Mas receptor, events that modulate vascular function. Taken together, the evidence suggests that PPARs regulate RAS participation. Additionally, RGZ exerts an antioxidant effect reducing DNA damage.

## Data Availability

The datasets generated during and/or analysed during the current study are available from the corresponding author on reasonable request.
